# To a new normal in radiation oncology: looking back and planning forward

**DOI:** 10.1177/0300891620962197

**Published:** 2020-10-12

**Authors:** Amelia Barcellini, Andrea Riccardo Filippi, Francesca Dal Mas, Lorenzo Cobianchi, Renzo Corvò, Pat Price, Ester Orlandi

**Affiliations:** 1 National Center of Oncological Hadrontherapy (Fondazione CNAO), Pavia, Italy; 2 Department of Radiation Oncology, IRCCS Policlinico San Matteo Foundation and University of Pavia, Pavia, Italy; 3 Lincoln International Business School, University of Lincoln, UK; 4 General Surgery Clinic, IRCCS Policlinico San Matteo Foundation, and Department of Diagnostic and Pediatric Science, University of Pavia, Pavia, Italy; 5 Department of Radiation Oncology, IRCCS Ospedale Policlinico San Martino, and Department of Health Science, University of Genoa, Genoa, Italy; 6 Department of Surgery and Cancer, Imperial College London, London, UK

The coronavirus disease 2019 (COVID-19) pandemic stands as one of the biggest crises of our time. COVID-19 massively impacts the economy, society, and the entire healthcare sector worldwide, requiring rapid reorganization of existing protocols and procedures.^1^ Most healthcare professionals had to change or adjust their roles, deployed to work on wards and intensive care units to take care of patients with COVID-19. Even departments not directly affected, like those dedicated to cancer care, had to rearrange their planned activity, limiting it to urgent cases in need of oncologic treatments.^2^ In some countries, “COVID-19–free” cancer hubs were instituted to guarantee safe, timely, and efficient diagnosis and treatment. However, it became evident that because of asymptomatic carriers who could spread the disease, such a definition is at best misleading. As a consequence, unmet medical needs have arisen, with the entire healthcare system searching for a compromise between the traditional patient-centered ethics to public health ethics.^3^

Oncologists are confronted with the difficult choice between protecting their patients by assuring maximal social distancing to reduce the risk of contagion versus maintaining adherence to the most effective schedules of cancer treatment. In radiation oncology, this challenge has been reconciled through a multipronged approach; a triage system has been established to guarantee timely, safe, and accessible treatments, ensuring at the same time the enforced social distancing.^4–6^ To respond to this challenge, in April 2020 a Global Coalition for Radiotherapy was launched^7^ bringing together radiotherapy (RT) professionals, industry, societies, and researchers worldwide to share experience and develop solutions.^8^

The recent literature has recognized the presence of three distinct phases in the COVID-19 era.^9^ The first phase is emergency, which is characterized by a series of contingency plans aimed at resilience to triage nonurgent cases, increase resources (both human and technical) to meet the needs of patients with COVID-19, and define new safety protocols for inpatient and outpatient services for all the people involved. In the absence of a vaccine or effective treatments, the current transition phase is characterized by the return of several clinical activities, coping at the same time with the presence of the virus. The transition phase borrows some practices and lessons learned during the emergency phase. The healthcare ecosystem is now starting to plan for the recovery phase, in a post–COVID-19 new normal. The recovery phase will need to redefine its protocols and procedures informed from the previous two stages. Learning from the COVID-19 pandemic, healthcare systems are aiming to become better prepared, developing what the literature calls antifragile strategies.^9,10^ Starting from the framework of Cobianchi et al.,^9^ we aim to highlight the main topics and issues to be addressed in the full recovery phase, based on the lessons learned from the previous stages of the COVID-19 era.

The main results are summarized in Figure 1.

**Figure 1.**
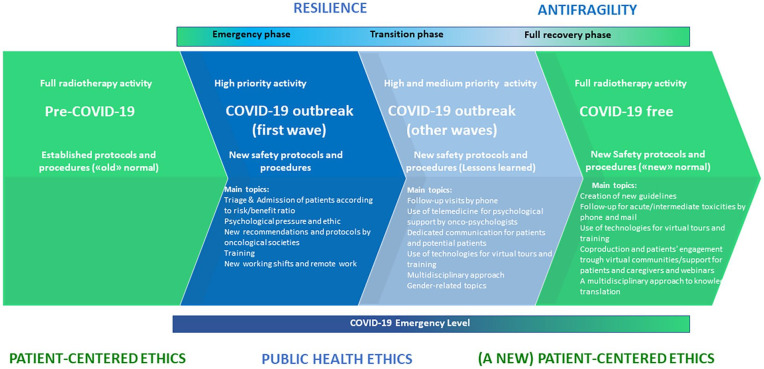
Radiotherapy in the coronavirus disease 2019 (COVID-19) era (adapted from Cobianchi et al.,^9^ 2020).

## Emergency phase: time for resilience

### Impact on patient care

The multidisciplinary management of patients rapidly adjusted to remote access through web-based technologies to share knowledge and discuss clinical cases. From the inception of the pandemic, adherence to RT treatments, often requiring several weeks of daily visits, emerged as an obvious challenge. RT plays a crucial role in oncologic treatment worldwide. Since the beginning of the COVID-19 outbreak, many different groups in China, Europe, and later all over the world started to discuss how to guarantee access to radiation oncology facilities while minimizing the risk of infection for both patients and staff.^7^ The first efforts were dedicated to establishing triage criteria based on cost/benefit ratios, considering the tumour biology, performance status, age of the patient, comorbidities, impact of the deferral on the expected survival, and quality of life.^11–13^ Guidelines on the correct use of personal protective equipment (PPE), which had been strengthened to enable safe treatments also in case of asymptomatic carriers, represented a priority. Many RT departments had to reorganize their internal routes and treatment time schedules for infected or suspected patients. The second wave of indications touched the different tumour sites, with specific guidelines.^14–18^ In most of them, changes in fractionation regimens were advised on an individual basis and given the impact of the epidemic spread on single institutions.^4,5,19^ The first data describing changes in RT practice were published from Italian centres.^20^ Inevitably, enrollment into clinical trials slowed down to cope with the general emergency protocols.^21^ Social distancing requirements limited in-person visits to patients with suspected disease progression or severe postactinic toxicities. For most remaining cases, follow-up was managed by phone. Sometimes care was relocated to designated facilities near the patients’ residence. Severe psychological consequences arose in both patients and clinicians.^22,23^ Both a sense of vulnerability to COVID-19 and the condition of isolation during the pandemic competed with the importance of adherence to life-saving treatments among oncologic patients.^24^

### Impact on healthcare workers

To reduce risk of exposure, clinical shifts were reorganized, and a contingent strategy was adopted to replace any symptomatic healthcare worker. Remote work was implemented whenever possible and sometimes extended to remote RT planning.^25^ Web-based technologies enabled knowledge sharing within multidisciplinary teams and discussion of clinical cases. Training activities for residents, medical students, and other trainees were reorganized consistently, as well as general safety training for all the personnel involved. In the most affected areas, peer education on COVID-19 management was provided to all hospital personnel assigned to work on the front line.^26^ Most internships and educational visits to institutes and RT departments were cancelled or postponed.

These sudden changes in the approach to patient management and treatment decision-making tested the resilience of oncologists daily, resulting in a high incidence of burnout,^27^ as also reported in a recent survey on oncology physicians and nurses working in Wuhan, China.^28^ A high incidence of posttraumatic stress disorder was also observed among healthcare workers during the H7N9 avian flu pandemic.^29^ Healthcare workers faced dual stress from fear of infection and risk of becoming contagious to their loved ones. at the same time, the practice of medicine was deprived of nonverbal communication during clinical evaluation, an essential component of empathy, drastically limited by PPE. Pietrantonio and Garassino^30^ reported feelings of fear and anger, suggesting that interinstitutional support, the inclusion of patients’ advocacy organizations, and oncology associations could help resilience to these challenges.

## Transition phase: applying what was learned

The transition phase has inherited some unsolved ethical and operational issues from the emergency phase. While still coping with the presence of COVID-19, a call for action concerns a rapid response to patient psychological stress,^31^ mainly due to the fear and awareness of infection and social isolation consequences (a well-known mortality risk for patients with cancer^32,33^). Patients were often negatively affected by the sudden change in the relationship with their clinicians and the concurrent lack of psychosocial support, due to limited contact with family members and friends and clinical areas.

Thus psychosocial support to patients undergoing oncologic therapies has emerged as a priority for the transition phase. Strategies include psycho-oncologist visits by telemedicine through e-meeting platforms. Webinars and books have been adopted to entertain patients in waiting rooms, since the need for sanitation of treatment rooms and clinical offices has introduced delays in care. The need for knowledge translation and sharing to be accessible to a layperson^34^ has been enhanced through official websites and social media channels, including messages to comfort and reassure patients, to decrease fear of infection, and to improve adherence to prescribed cancer treatments. Many radiation technicians and healthcare professionals worldwide started writing their names or putting their pictures on white coats to reduce psychological distance during treatments.

From a clinical perspective, the practices introduced from the previous emergency phase are sustained during the transition phase. Some have proposed a change in standard RT procedures that may remain in coming years. For instance, the use of telemedicine for imaging exchange or remote planning has proven to be a robust and efficient care solution, likely to be maintained whenever feasible and clinically appropriate after full recovery.

Clinical experience was already available on safety and efficacy of converting most standard fractionation schedules into shorter, hypofractionated ones.^16,31,35^ The COVID-19 pandemic inevitably precipitated the widespread adoption of hypofractionated regimens, with need of acquiring long-term morbidity data on safety and efficacy after this initial experience.

## Full recovery phase: proposals for a new normal

The emergency and transition phase raised several challenges for RT. Regarding official guidelines from scientific societies, some open questions emerge, and the main goal is maximizing the benefit produced during the previous phases of the outbreak.

Institutes all over the world will need to decide which lessons learned from the first two phases may become standards for the new normal. Institutes will need to stick to international recommendations and their strategic outcomes, looking back, and planning forward. Longer follow-up is needed to learn the long-term effects of RT in patients treated with hypofractionated regimens for specific cancer types and settings, and compare them to those reported for conventional regimens. Adherence and compliance studies to measure the safety and effectiveness of follow-up visits by phone are warranted. Specifically, the impact on timely detection of recurrences suitable for salvage therapies needs to be balanced versus the advantages of fewer visits (both for patients and health workers).

During the emergency phase, the paradigm of treatments changed to a minimalistic approach, reserving treatment to urgent or indispensable conditions, based on common sense and evidence-based medicine. The long-term results need to be evaluated carefully to inform new models of medical care.^3^ A database of patients treated in the COVID-19 era should be required in each RT department to evaluate the results of the changes adopted for certain tumours during the pandemic. The latter issue is important because, in some settings, standard cancer care was compromised, because it was not possible to combine chemotherapy, targeted therapies, or immunotherapy with RT according to established guidelines.

The same informational technology adopted during the pandemic can be applied to improve patient databases and reduce waiting list time. The system can be enhanced and monitored using data gathered through appropriate information technology tools and control systems.

The use of telemedicine should be encouraged as a way to engage patients and foster coproduction.^36^ New technologies allow monitoring patients’ conditions, can provide psychological support through virtual communities, and help to disseminate information about complex treatments. Webinars and virtual tours could be used to share and disseminate knowledge about RT among the general public, general practitioners, and other healthcare professionals. National and international scientific societies could take advantage of webinars and interactive lessons to receive updates (regarding new clinical trials or new radiation technologies). Even if the classical in-person visit with a physical examination cannot always be replaced, some of the follow-up visits can. Physical meeting is a crucial aspect of team science, but the same e-meeting platforms and tools used among multidisciplinary teams in the emergency and transition phases can be re-purposed to sustain communication among overseas institutes for clinical, educational, and research purposes, saving time and travel expenses.^37^ Telemedicine can also facilitate second opinion visits. Remote RT planning, proved to be secure and useful,^25^ could also be implemented to reduce commuting and better balance work and private life, especially for healthcare workers with young children. One must keep in mind that permanently implementing these new clinical procedures and adopting a new professional daily life with more distancing in human relationships, especially in the physician–patient interaction, will have unpredictable consequences.

The wellbeing of clinical staff can be enhanced by reorganizing shifts and promoting telework whenever possible. These changes may reduce the gender gap and promote equality. Moreover, the current practice of tumor-specific oncology through dedicated interdisciplinary teams can continue in the new normal, and continue to enhance knowledge, sharing the expertise of each subspecialty, including emergency management.

Educational opportunities have arisen for the new generation of radiation oncologists, encompassing novel skills beyond those relevant for radiation oncology, including soft skills like team building, emergency and change management, and leadership, to better coordinate teams and integrate radiation oncology care with other medical specialties, like emergency room medicine and infectious diseases.

The new normal will require a significant effort from scientific societies and institutes all over the world to join forces to share and translate knowledge with a multidisciplinary approach to get the best out of the first two phases of the COVID-19 crisis. Considering that the measures to control the spread of COVID-19 have probably postponed diagnosis and treatment, affecting prognosis, rapid and open communication of experience should develop around the world^7^ to share data about the outcome of patients treated during the previous phases and to evaluate the effect of potential delay of diagnosis and treatment for oncologic patients. Moreover, it could be important to report and record the impact of COVID-19 on the clinical trial. The lessons learned must represent the basis for a more robust RT system, leading to renewed ethics towards quality and safety for patients and the RT professionals.^3,22,23,38,39^
